# Exercising in Hypoxia and Other Stimuli: Heart Rate Variability and Ventilatory Oscillations

**DOI:** 10.3390/life11070625

**Published:** 2021-06-28

**Authors:** Eric Hermand, François J. Lhuissier, Aurélien Pichon, Nicolas Voituron, Jean-Paul Richalet

**Affiliations:** 1 ULR 7369-URePSSS-Unité de Recherche Pluridisciplinaire Sport Santé Société, Univ. Littoral Côte d’Opale, Univ. Artois, Univ. Lille, 59379 Dunkerque, France; 2 UMR INSERM U1272 Hypoxie & Poumon, Université Sorbonne Paris Nord, 93017 Bobigny, France; francois.lhuissier@aphp.fr (F.J.L.); nicolas.voituron@univ-paris13.fr (N.V.); richalet@univ-paris13.fr (J.-P.R.); 3 Médecine de L’Exercice et du Sport, Assistance Publique Hôpitaux de Paris Hôpital Jean Verdier, 93140 Bondy, France; 4 Laboratoire MOVE (EA6314), Université de Poitiers, 86073 Poitiers, France; aurelien.pichon@univ-poitiers.fr; 5 Département STAPS, Université Sorbonne Paris Nord, 93000 Bobigny, France

**Keywords:** hypoxia, exercise, periodic breathing, heart rate variability, hypercapnia, hyperoxia, acetazolamide, mathematical modeling

## Abstract

Periodic breathing is a respiratory phenomenon frequently observed in patients with heart failure and in normal subjects sleeping at high altitude. However, until recently, periodic breathing has not been studied in wakefulness and during exercise. This review relates the latest findings describing this ventilatory disorder when a healthy subject is submitted to simultaneous physiological (exercise) and environmental (hypoxia, hyperoxia, hypercapnia) or pharmacological (acetazolamide) stimuli. Preliminary studies have unveiled fundamental physiological mechanisms related to the genesis of periodic breathing characterized by a shorter period than those observed in patients (11~12 vs. 30~60 s). A mathematical model of the respiratory system functioning under the aforementioned stressors corroborated these data and pointed out other parameters, such as dead space, later confirmed in further research protocols. Finally, a cardiorespiratory interdependence between ventilatory oscillations and heart rate variability in the low frequency band may partly explain the origin of the augmented sympathetic activation at exercise in hypoxia. These nonlinear instabilities highlight the intrinsic “homeodynamic” system that allows any living organism to adapt, to a certain extent, to permanent environmental and internal perturbations.

Cardiovascular and respiratory systems are two distinct systems fulfilling different and complementary missions. Hence, to meet the metabolic demands of the body, they have to function together, as a whole, through multiple and complex cardiorespiratory coupling. Moreover, they must adapt their own functioning and their relationship when the organism is confronted by environmental and/or physiological stressors. These adaptations may lead to transient or chronic oscillatory outputs in both systems, observed in normal subjects or in patients. Recent technological and mathematical advances allow a better characterization and a fine-tuned understanding of these oscillatory physiological behaviors. The present work, ensuing successive protocols carried out by our research group, aims to review these specific mechanisms occurring in healthy humans exercising in hypoxia and other various stressors, such as hypercapnia and hyperoxia.

## 1. Heart Rate Variability

### 1.1. HRV: What and Why?

**Figure 1:**
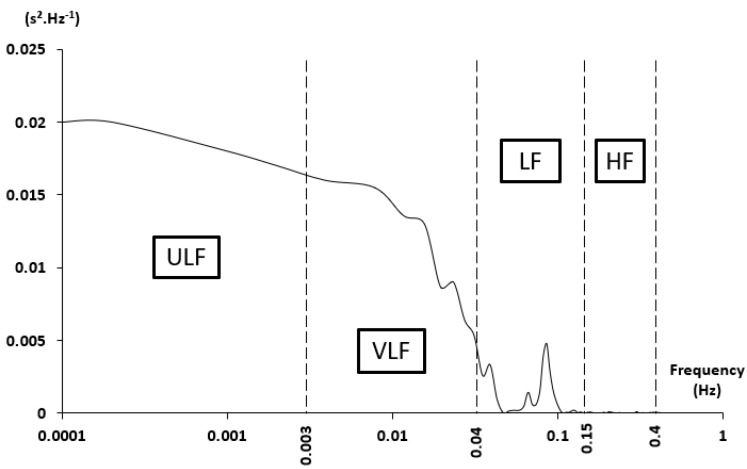
Example of an IBI spectral density (logarithmic scale), including the different bands: Ultra-low, very low, low, and high frequencies (ULF, VLF, LF, and HF, respectively). Extracted from [7].

Heart rate variability (HRV) is now a widely used tool to provide valuable information about cardiorespiratory status and health in a large range of subjects, from elite athletes [1] to heart failure patients [2]. Briefly, a reduced HRV illustrates a cardiovascular system under stress or submitted to a pronounced fatigue [3,4]. Whereas an ECG signal is required for HRV accuracy, a spectral analysis of the inter-beat interval (IBI) remains, among various signal-processing methods, a reliable tool for the measurement of the sympathovagal balance between sympathetic (SNS) and parasympathetic (PNS) nervous systems [5,6]. The obtained spectral signal is divided in successive frequency bands in normal subjects (Figure 1): 0–0.0033 Hz ultra-low frequency (ULF), 0.0033–0.04 Hz very low frequency (VLF), 0.04–0.15 Hz low frequency (LF), and 0.15–0.4 Hz high frequency (HF). Usual HRV parameters computed from these bands are VLF, LF, and HF powers (ms²); LF/HF power ratio (LF peak power)/(LF power) ratio; and total spectral power (ms^2^). Additional parameters may also be added, such as the frequency of the peak in each HRV band.

### 1.2. HRV: Methodological Aspects

Physiological significations of HRV bands are yet to be totally comprehended. For example, ULF band is not fully understood but might be driven by complex interactions between circadian rhythm, core body temperature, metabolism, and the renin-angiotensin system [8], and is therefore scarcely used in clinical practice. There is currently no consensus about the contribution of SNS and PNS on this ULF band. Physiological mechanisms of the VLF band are also yet to be determined, although PNS might play a central role in its power [9], along with physical activity, thermoregulation, and the renin-angiotensin system [10]. The LF band is the result of both PNS and SNS activity, but the SNS does not produce much activity above 0.1 Hz, while the PNS may affect heart rhythm down to 0.05 Hz. At rest, the LF band mainly reflects baroreflex activity and not cardiac sympathetic innervation [8]. Finally, the HF band reflects parasympathetic activity, but also shows a cardiorespiratory coupling, named respiratory sinus arrhythmia [11].

## 2. Ventilatory Oscillations at Exercise in Hypoxia

**Figure 2:**
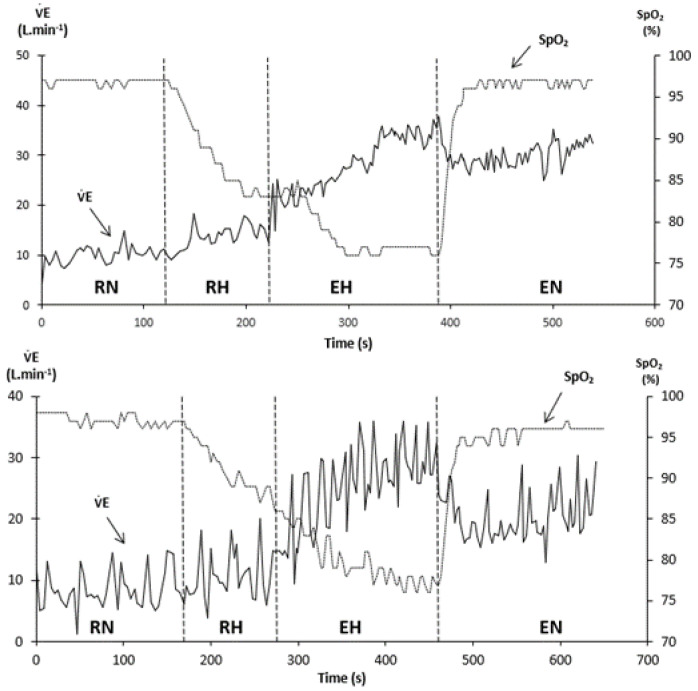
Breath-by-breath output of minute ventilation ($\dot{V}$E), pulse O_2_ saturation (SpO_2_), and end tidal PCO_2_ (PETCO_2_) during a hypoxia exercise test. Lower panel: Subject with a high HVRe (0.93 L.min^−1^.kg^−1^). Upper panel: Subject with a low HVRe (0.60 L.min^−1^.kg^−1^). From [17].

**Figure 3:**
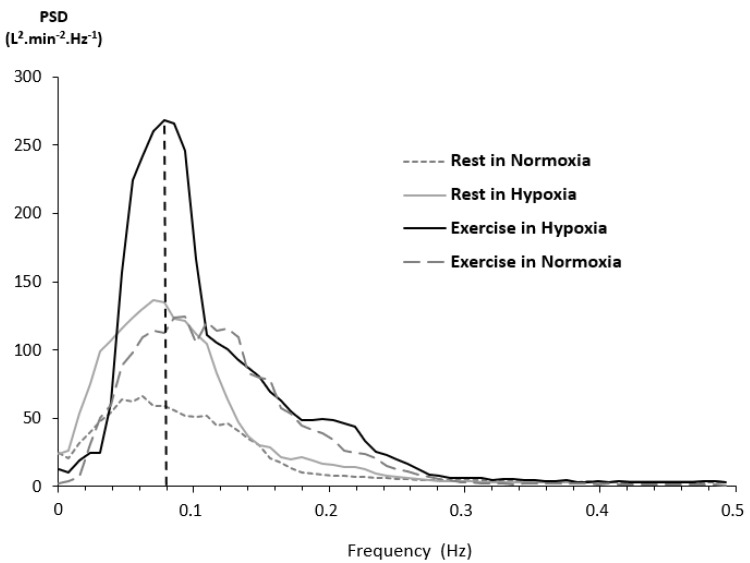
Example of spectral analysis of minute ventilation by Fast Fourier Transform in the 4 phases of a hypoxia exercise test. RN: Rest in normoxia, RH: Rest in hypoxia, EH: Exercise in hypoxia, EN: Exercise in normoxia. PSD: Power Spectral Density. Note that the frequency of the main peak in hypoxic exercise conditions (EH) is around 0.08 Hz, which corresponds to a period of 12.5 s. From [17].

**Figure 4:**
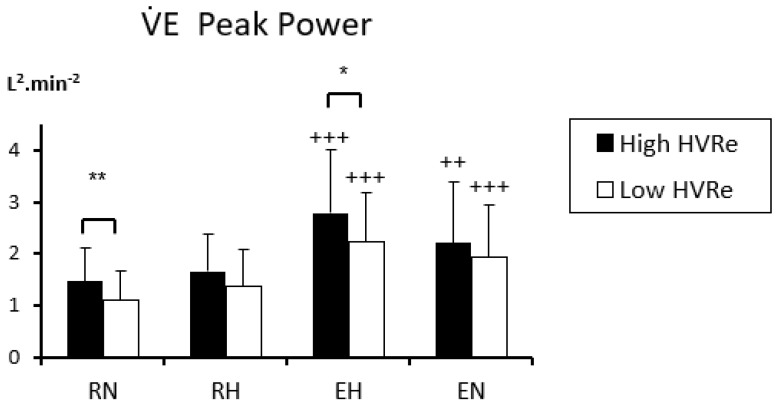
$\dot{V}$E power of the main peak obtained from the spectral analysis of minute ventilation during the various phases of a hypoxia exercise test. RN: Rest in normoxia, RH: Rest in hypoxia, EH: Exercise in hypoxia, EN: Exercise in normoxia. Mean ± SD. Condition vs RN: ++, *p* < 0.01, +++, *p* < 0.001. High HVRe vs. low HVRe: *, *p* < 0.05; **, *p* < 0.01. From [17].

As HRV represents a key tool in assessing numerous parameters in healthy and pathological conditions, there is growing evidence about the relevance of analyzing its ventilatory counterpart. For example, periodic breathing and/or ventilatory disorders are frequent in chronic heart failure (CHF) patients [12], and periodic breathing observed in exercising CHF patients is associated with a negative prognosis [13,14]. Periodic breathing is also observed in normal subjects sleeping at high altitude. However, these ventilatory disorders have not been studied in healthy subjects exercising in hypoxia until recently. A first observation unveiled a marked periodic breathing in a single middle-aged climber during a mild effort at 5533 m [15,16]. Based on a Fast Fourier Transform of breath-by-breath ventilation signals (not smoothed over 30-s intervals, Figure 2), a laboratory-controlled study evidenced periodic breathing in healthy subjects exercising in hypoxia, and allowed more extensive description and understanding of the underlying physiological mechanisms [17]. First, the period of ventilatory oscillations is much shorter than the one observed in heart failure or sleep apnea syndrome (SAS; 11~12 s vs. 30~60 s (Figure 3)) [18,19]. Second, this period is negatively correlated with cardiac output ($\dot{Q}$c) and ventilation ($\dot{V}$E): The higher the exercise intensity, the shorter the period. Third, the power of ventilatory oscillations (e.g., their amplitude), is positively correlated to $\dot{Q}$c and $\dot{V}$E, as ventilatory oscillations are larger with high exercise intensity. Fourth, greater respiratory oscillations are associated with a greater chemosensitivity to hypoxia, meaning that subjects exhibiting a higher ventilatory response to hypoxia (HVRe) produce greater ventilatory oscillations (Figure 4).

These observations point out the key role of peripheral chemoreceptors in the genesis of breathing instability, triggered by simultaneous environmental (hypoxia) and physiological (exercise) stressors. It is also interesting to note that an increase in cardiac output (during exercise) exacerbates the oscillations. In contrast, in CHF patients, the predominant hypothesis to explain the Cheyne-Stokes respiration (CSR) pattern is an increase in circulation time delay due to low cardiac output [20].

## 3. Other Factors Impacting Exercise Oscillatory Ventilation

If a hypoxic environment can destabilize the breathing control system during mild exercise, what are the effect(s) of other environmental and pharmacological stimuli?

### 3.1. Hypercapnia

Chemosensitivity to hypoxia underlines the role of peripheral chemoreceptors (mainly carotid bodies) in ventilatory oscillations, but what about the contribution of central chemoreceptors, illustrated by the ventilatory response to CO_2_ (HCVR)?

**Figure 5:**
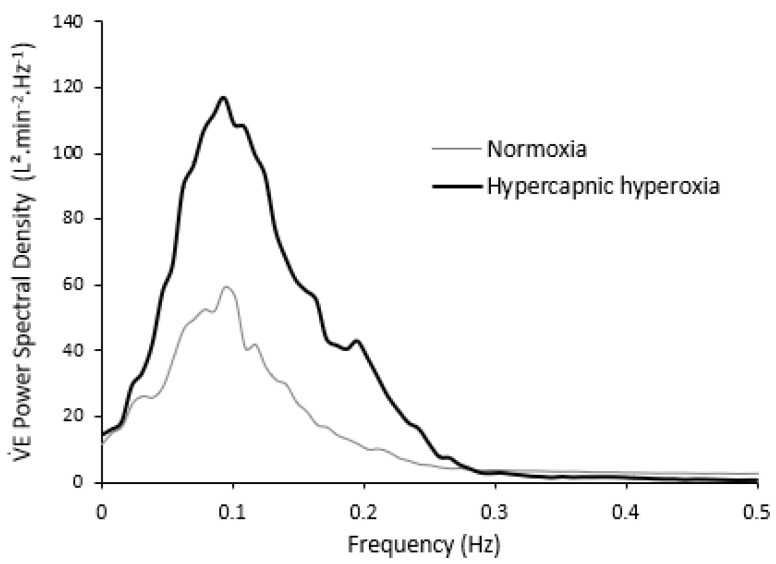
Power spectral density of the ventilation signal at exercise: Normoxia and hypercapnic hyperoxia. From [20].

In CHF patients, CO_2_ inhalation during sleep and exercise considerably diminishes apnea occurrences in CSR by keeping CO_2_ arterial pressure above the apneic threshold [21,22,23]. In healthy subjects, the opposite phenomenon is observed: Hyperoxic hypercapnia deepens respiratory oscillations during exercise, with a similar period (Figure 5) [24]. Other relationships between $\dot{V}$E power or period and physiological parameters ($\dot{Q}$c, $\dot{V}$E) are preserved, as observed in hypoxic condition. 

The involved mechanisms are still debated. If hyperoxia silences the activation of peripheral chemoreceptors [25], CO_2_ is known to stimulate central chemoreceptors, although it is also, to a lesser extent, a determinant factor in peripheral chemoreflex activation in hypoxia [26]. Therefore, the noticeable increase in ventilatory oscillations in hypercapnia could be mainly attributed to the activation of central chemoreceptors.

### 3.2. Hyperoxia

**Figure 6:**
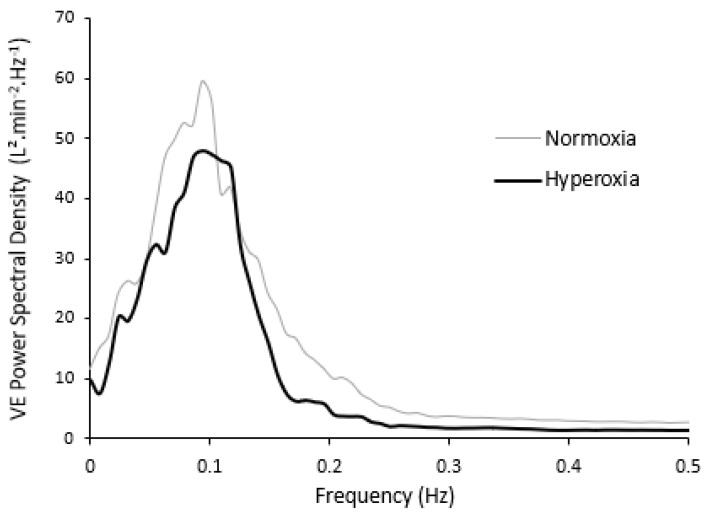
Power spectral density of the ventilation signal at exercise: Normoxia and hyperoxia. From [24].

Breathing in pure O_2_ does not elicit a ventilatory response mediated by chemoreceptors activation [27]. If hyperoxia presumably silences the activation of peripheral chemoreceptors, unlike CO_2_ inhalation, hyperoxia has more conflicting effects in humans. Hyperoxia reduces the hypoxemia involved in the mechanisms of periodic breathing, and therefore improves heart condition, physical performance, and apnea/hypopnea index in CSR patients [28,29], but does not abolish ventilatory oscillations in obstructive sleep apnea [30] or CHF patients [31]. Conversely, at high altitude, restoring a normal arterial O_2_ pressure by O_2_ inhalation significantly reduces sleep apnea and periodic breathing and improves sleep quality in normal subjects [32,33]. As expected, in healthy subjects exercising in hypoxia, no further breathing instability was observed (Figure 6) [24].

### 3.3. Acetazolamide (ACZ)

**Figure 7:**
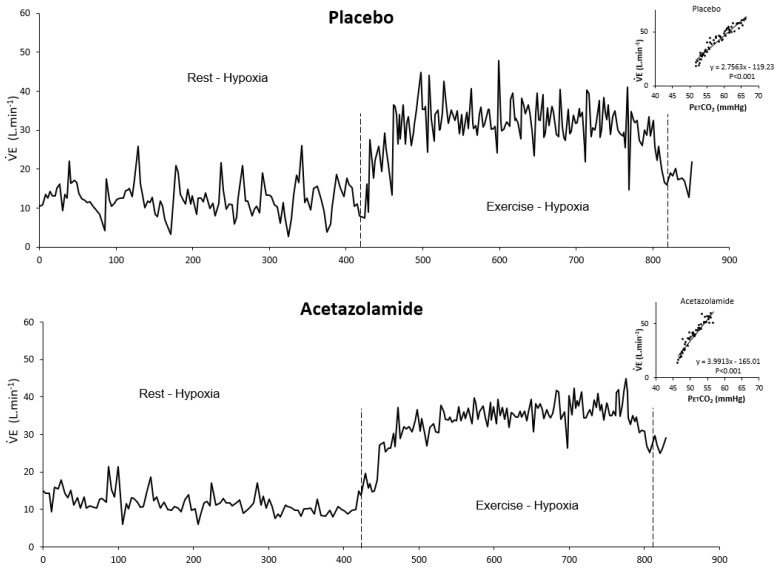
Example of breath-by-breath ventilation recordings under placebo (upper panel) and acetazolamide (lower panel) treatment. Inlets: Ventilatory response to CO_2_ (upper right: Placebo, lower right: Acetazolamide). From [24].

ACZ is mainly used in the prevention of acute and chronic mountain sickness in altitude [34,35,36] but also to lower the occurrence of periodic breathing during sleep in altitude [37] and CHF patients [23]. ACZ complex action is yet to be fully understood, and involves several contradictory mechanisms, including a stimulation of central chemoreceptor and a reduction of peripheral chemoreceptors activity [38,39]. A stimulation of central chemoreceptors by CO_2_ or acetazolamide (ACZ) elevates ventilation level and arterial O_2_ saturation at high altitude and considerably reduces occurrence and length of apnea episodes. Likewise, a similar effect on ventilatory oscillations was observed in awake CHF patients at rest and during exercise [23,40]. In normal subjects, we found a similar effect of ACZ on $\dot{V}$E oscillations at exercise in hypoxia: The ventilation level was augmented, but breathing instability was considerably reduced (Figure 7) [24]. These data raise further interrogations about the action of ACZ on the breathing control system. ACZ inhibits the peripheral effect of hypoxia [19] and enhances the central effect of hypercapnia on ventilation [41]. However, as a result, ventilatory oscillations are blunted, confirming the key role of the activity of peripheral chemoreceptors in the genesis of ventilatory oscillations.

### 3.4. Dead Space

**Figure 8:**
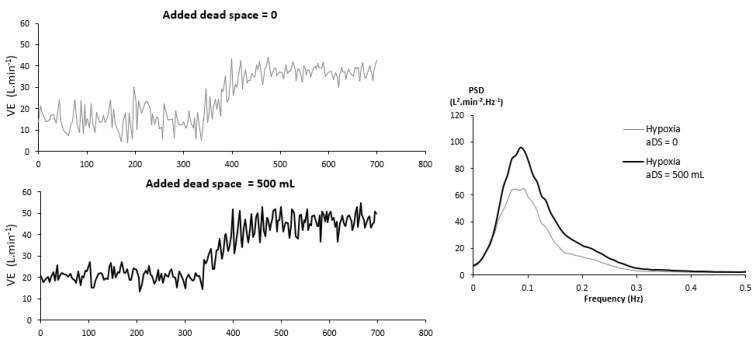
Left panel: Example of breath-by-breath ventilation recordings in hypoxia without and with added dead space (respectively upper and lower panels). Right panel: Power spectral densities of ventilation signal at exercise in 2 conditions: Hypoxia without added dead space (aDS) and hypoxia with aDS. From [48]. Copyright permission has been obtained from Elsevier.

Except environmental or pharmacological interventions, an artificial modification in upper airways might impact the breathing control system, for example, by adding an external dead space. This method has proven to be very efficient in drastically reducing periodic breathing in patients [42,43,44] and in normal subjects at high altitude [45,46]. By maintaining a relatively high arterial pressure in CO_2_ (PaCO_2_) combined with a higher $\dot{V}$E, this method prevents PaCO_2_ from falling below the apnea threshold and the subsequent genesis of a central apnea cycle [47]. In healthy subjects exercising in hypoxia, in contrast with the aforementioned data, adding a dead space exacerbates ventilatory oscillations (Figure 8) [48]. The involved mechanisms could be similar to CO_2_ inhalation. Whereas hypercapnia and added dead space reduce $\dot{V}$E oscillations in central and obstructive sleep apnea by keeping PaCO_2_ above apnea threshold, the subsequent augmented $\dot{V}$E level (due to hypercapnia and added dead space) maintains an aggravated destabilization of respiratory control system in exercising subjects in hypoxia.

### 3.5. Summary

**Figure 9:**
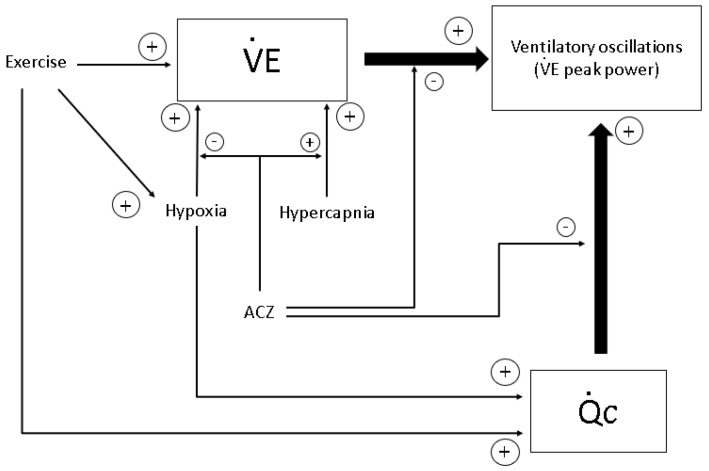
Schematic diagram of potential mechanisms involved in the genesis of ventilatory oscillations in normal subjects at exercise. Breathing instability is directly related to the intensity of $\dot{V}$E and $\dot{Q}$c. Exercise, hypoxia, and hypercapnia increase $\dot{V}$E and $\dot{Q}$c, and therefore increase ventilatory oscillations. ACZ inhibits the effect of hypoxia and enhances the effect of hypercapnia on ventilation and blunts the relation between $\dot{V}$E or $\dot{Q}$c and ventilatory oscillations. From [24].

The mechanisms involved in ventilatory oscillations in healthy subjects at exercise in various environmental and/or pharmacological conditions are illustrated in Figure 9. The activity of the respiratory central pattern generator could be modulated by an oscillator, depending on different chemical stimuli, and would therefore promote the instability of the system, directly related to the level of $\dot{V}$E and $\dot{Q}$c. Thus, it is not surprising to observe the destabilizing effect of exercise, hypoxia, and hypercapnia, which are also known to increase $\dot{V}$E and $\dot{Q}$c. ACZ has contrasting effects: It reduces the effect of hypoxia on ventilatory oscillations and augments the effect of hypercapnia on $\dot{V}$E, but also blunts the relation between ventilatory oscillations and $\dot{V}$E or $\dot{Q}$c. Finally, hyperoxia deactivates peripheral chemoreceptors [49], and, to our knowledge, has no action on central chemoreceptors in humans [50]. The period of these $\dot{V}$E oscillations remains between 11 and 12 s in each of these various environmental conditions, and, hence, is notably shorter than those observed in CHF patients.

## 4. Mathematical Modeling of Breathing Control System: A Two-Way Process between Theory and Observation

The successive attempts of modeling the ventilatory control system were based on the mass balance equation for O_2_ and CO_2_ [51]. Whereas most models have focused on steady-state breathing under hypoxia and/or hypercapnia, only a few have covered the complex topic of instability of ventilation control, in SAS and CHF [52,53]. Models became progressively more complex with the addition of numerous cardiorespiratory and neural inputs and the progress in computing science [54]. These simulations have brought valuable clinical insights in our understanding of breathing disorders in SAS and CHF patients, both in their intrinsic mechanisms and in the potential treatments by O_2_ or CO_2_ inhalation [20].

**Figure 10:**
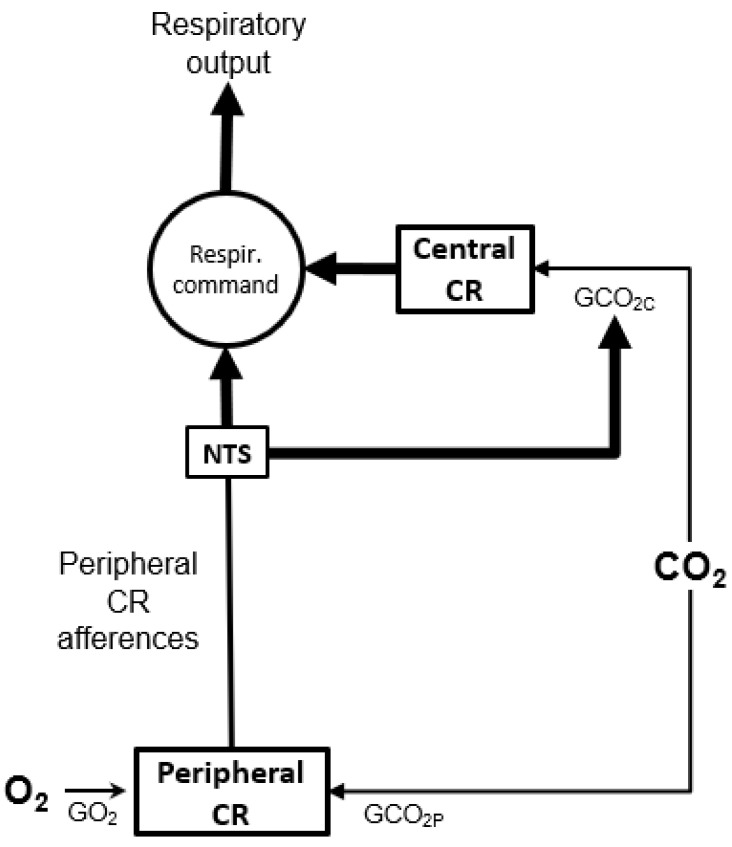
Diagrams of a central/peripheral interactive model of ventilation control system. The central respiratory command receives direct or indirect afferences from peripheral (through nucleus tractus solitaries, NTS) and central chemoreceptors (PCR and CCR, respectively), modulated by PaO_2_ and PaCO_2_. Hence, the level of hypoxia and hypercapnia acts on respiratory outputs via O_2_ gain (peripheral GO_2_, as a ventilatory response to a change of FIO_2_) and CO_2_ gain (peripheral GCO_2P_ and central GCO_2C_, to a change of FICO2). From [56]. Copyright permission has been obtained from Elsevier.

**Figure 11:**
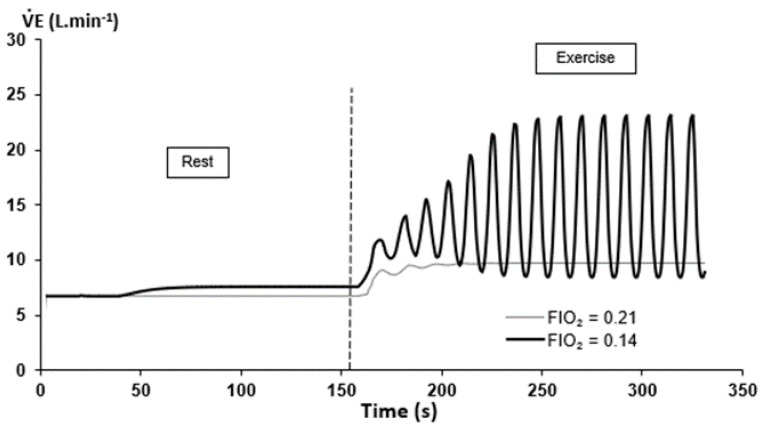
Variation of $\dot{V}$E for 2 levels of FIO_2_ in the additive model: Normoxia (FIO_2_ = 0.21) and hypoxia (FIO_2_ = 0.14). From [56]. Copyright permission has been obtained from Elsevier.

**Figure 12:**
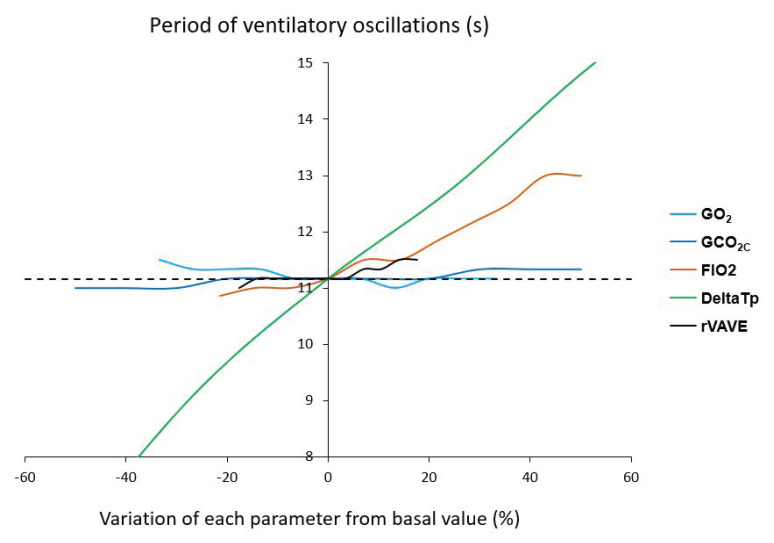
Period of ventilatory oscillations according to several cardiorespiratory variables from their nominal values: Peripheral O_2_ gain (GO_2_), central CO_2_ gain (GCO_2C_), inhaled fraction of O_2_ (FIO_2_), delay of blood convection from lung to carotid bodies (DeltaTp), arterial O_2_ partial pressure at SaO_2_ = 50% (P50), total lung capacity (TLC), and alveolar/total ventilation ratio (rVAVE). From [56]. Copyright permission has been obtained from Elsevier.

**Figure 13:**
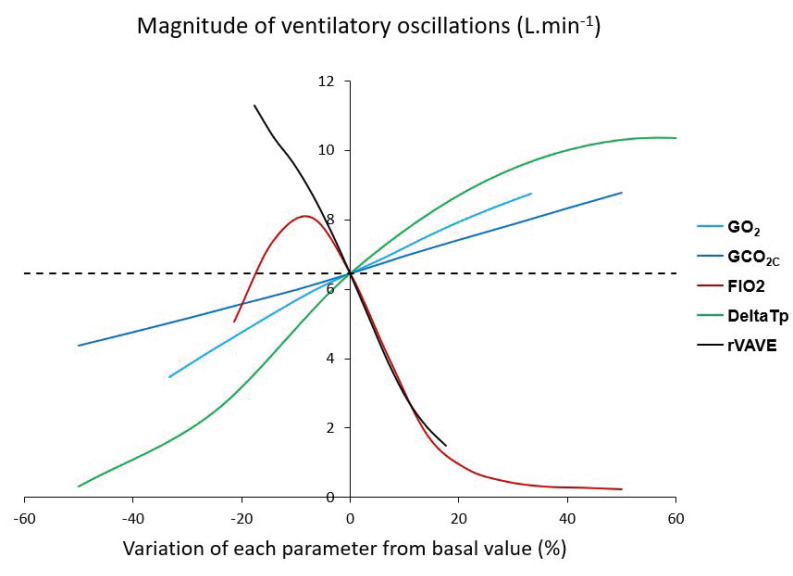
Magnitude of ventilatory oscillations according to several cardiorespiratory variables from their nominal values: Peripheral O_2_ gain (GO_2_), central CO_2_ gain (GCO_2C_), inhaled fraction of O_2_ (FIO_2_), delay of blood convection from lung to carotid bodies (DeltaTp), arterial O_2_ partial pressure at SaO_2_ = 50% (P50), total lung capacity (TLC), and alveolar/total ventilation ratio (rVAVE). Magnitude mainly increases with GCO_2C_, GO_2_ (in a nearly linear manner), and DeltaTp and decreases with P50, rVAVE, TLC, and FIO_2_. From [56]. Copyright permission has been obtained from Elsevier.

Research in the last decades, in various animal model and afference-isolated preparations of chemoreceptors, has unveiled the tight interdependence between central and peripheral chemoreceptors [55]. In normal human subjects, the aforementioned preliminary studies have unveiled the complex interplay between O_2_ and CO_2_ sensors (e.g., peripheral and central chemoreceptors, Figure 10) leading to ventilatory instability when submitted to concomitant environmental (hypoxia, hypercapnia, hyperoxia), pharmacological (ACZ), and physiological (exercise) stimuli [17,24]. During exercise, this instability related to $\dot{Q}$c, $\dot{V}$E, HVRe, and HCVR was not included in the existing models until recently. Several mathematical models were created considering the effects of O_2_ and CO_2_ on chemoreceptors, but also the interaction between O_2_ and CO_2_. Various parameters were introduced in the models: Fraction of inspired O_2_ (FIO_2_), O_2_ and CO_2_ chemoreceptor gains, blood convection delay from lungs to chemoreceptors (ie $\dot{Q}$c), lung capacity, and dead space volume [56]. First, simulations showed that the output of the respiratory control system becomes oscillatory under simultaneous physiological and environmental stressors (exercise and hypoxia, Figure 11). Second, the period of $\dot{V}$E oscillations remains between 10 and 15 s and is mainly correlated to the level of hypoxia and the convection delay between lungs and peripheral chemoreceptors (Figure 12). Finally, more factors are potentially able to modify the power of $\dot{V}$E oscillations, the most important being FIO_2_, the O_2_ and CO_2_ gains, the convection delay from lung to peripheral chemoreceptors, and the dead space volume (Figure 13). 

On one hand, this mathematical modeling confirmed the field observations about the effects of hypoxia and exercise on $\dot{V}$E pattern (oscillatory period and amplitude) and on parameters altering or enhancing ventilatory instability [17,24]. On the other hand, these simulations pointed out other factors able to influence this instability, such as dead space, which was later confirmed in a dedicated protocol [48]. In the future, an enhanced mathematical model could be tested on other pathological conditions at exercise, such as heart failure patients, in whom cardiac function is impaired, whereas chemosensitivity to CO_2_ is increased.

## 5. HRV and Ventilatory Oscillations at Exercise in Hypoxia: What Relationship?

The relationship between HRV and periodic breathing has been studied mainly in severe CHF patients, in which pronounced central or mixed apnea are correlated with a lower cardiac output [57,58], as opposed to healthy subjects exercising in hypoxia. This illustrates the role of the autonomic nervous system (ANS) in the complex cardiorespiratory control system in a subject submitted to various stressors (physiological, environmental, etc.). Therefore, it highlights a tight link between cardiovascular and respiratory systems [59], although the underlying mechanisms are still yet to be fully understood. First, hypoxia generally induces a lower overall HRV and a higher HR [60]. A lower LF power is counterbalanced by an even greater withdrawal in the HF band, which may be due to a sympathetic predominance [61]. Second, exercise deeply alters HRV components by decreasing overall HRV, especially LF and HF power [62,63]. However, temporal relations between periodic breathing and HRV oscillations have not received much attention, except observations in healthy subjects exhibiting central sleep apneas during sleep at high altitude, where amplitudes of HRV parameters were correlated to respiratory amplitude [64]. 

**Figure 14:**
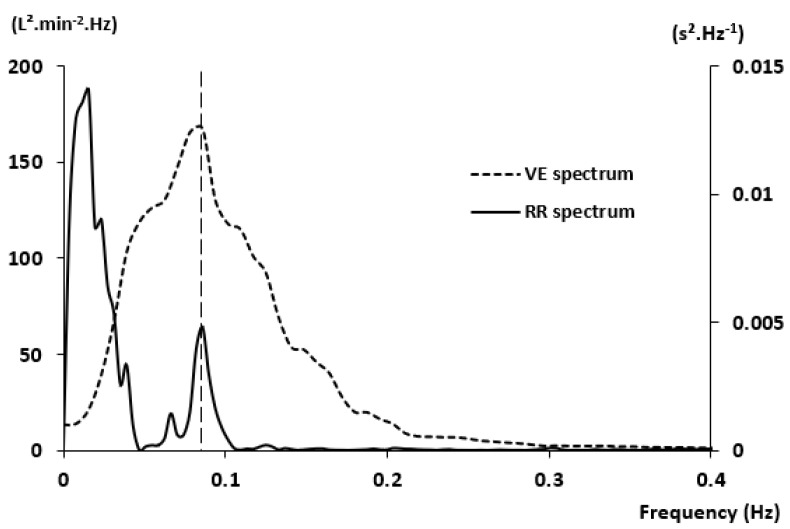
Spectrum analysis of a breath-by-breath ventilation recording and RR signal in hypoxia during exercise. From [7]. Copyright permission has been obtained from SPRINGER.

In normal subjects exercising in hypoxia, spectral analysis of both $\dot{V}$E and IBI signals evidenced a common peak at the same frequency (Figure 14), located in the LF band, at 0.085 Hz (ie 11~12 s) [7]. It also showed, despite an expected depressed overall HRV, that the rises of $\dot{V}$E peak power and HRV peak power are positively correlated: The greater the $\dot{V}$E oscillations, the greater the HRV oscillations. Moreover, the (LF peak power)/(LF power) ratio, illustrating the contribution of peak power on the LF band, indicates that more than one-third of LF power was concentrated in the LF peak and remained constant for almost all conditions. This might explain the origin of LF band between 0.04 Hz and 0.15 Hz where ventilatory and IBI oscillations occurred. In a larger clinical and physiological context, these data bring a new light on the origin of this peak in the LF band, which was unclear until lately. It could provide a novel insight on the physiological mechanisms in play in various pathological processes involving cardiorespiratory coupling, such as heart failure.

## 6. Conclusions and Perspectives

Until recently, the periodic phenomena of HRV and ventilation were distinctively investigated mostly in patients (heart failure, sleep apnea) and in subjects exposed to altitude, during sleep. The recent advances in fundamental knowledge helped to partially explain the complex interactions between respiratory and cardiovascular systems in normal subjects submitted to several stimuli (hypoxia and exercise). The observed oscillations in both signals may further illustrate the necessary adaptations to a new context, at a cellular and organism level, not too distant from the basal environment (as in CHF-related sleep apnea). A deeper reflection would even lead us to consider that variability/instability accommodation represents a crucial ability to maintain homeostasis in any new circumstances [65]. As an example, a new concept of “homeodynamics” was introduced during the 1990s, which pinpoints the dynamic properties of physiological regulation systems [66]: Nonlinear instabilities of biological variables trigger behavioral patterns that maintain a living organism in an alert stage when confronted to environmental or internal perturbations. Hypoxia, by essence variable in time and in localization in the body, illustrates this concept of homeodynamics that defines a living organism as a complex system in permanent instability, exposed to environmental and internal perturbations. Here would lay a condition for perpetuating life in a constantly changing ecosystem.

## Data Availability

The data presented in this study are available on request from the corresponding author. The data are not publicly available due to privacy reason.
